# 1166. The Cost-Effectiveness Evidence of Enhanced Influenza Vaccines in Adults ≥65 Years of Age: A Literature Review and Decision-Making Implications

**DOI:** 10.1093/ofid/ofad500.1006

**Published:** 2023-11-27

**Authors:** David FismanNorberto Giglio, Sergio Márquez-Peláez, Van Nguyen, Maarten Postma, Andrea Pugliese, Jesús Ruiz-Aragón, Analia urueña, Joaquin F Mould-Quevedo

**Affiliations:** Hospital de Niños de Buenos Aires, Belgrano, Ciudad Autonoma de Buenos Aires, Argentina; Pablo de Olavide University, Sevilla, Andalucia, Spain; VHN Consulting, Montreal, Quebec, Canada; Department of Health Sciences, University of Groningen, Groningen, Groningen, Netherlands; Department of Mathematics, University of Trento, Trento, Trentino-Alto Adige, Italy; Hospital de la Línea, Cadiz, Andalucia, Spain; Centro de Estudios para la Prevención y Control de Enfermedades Transmisibles, Isalud University, Buenos Aires, Argentina., Flores, Ciudad Autonoma de Buenos Aires, Argentina; CSL Seqirus Inc., Summit, New Jersey

## Abstract

**Background:**

Older individuals have high rates of seasonal influenza-related hospitalizations and deaths. For this population, enhanced vaccines have been designed to mitigate the effect of age-related immunosenescence by providing higher immunogenicity and increased relative vaccine effectiveness (rVE) compared with standard vaccines. The objective of this review is to assess the assumptions taken in published cost-effectiveness analyses (CEA) and analyze the results and decision-making implications on enhanced influenza vaccines for older adults (65yrs+).

**Methods:**

A targeted literature review was performed to identify economic evaluations of two enhanced vaccines in older individuals: adjuvanted trivalent/quadrivalent vaccine (aTIV/aQIV) and high-dose trivalent/quadrivalent vaccine (HD-TIV/HD-QIV). PubMed was searched for publications in March 2023, limited to studies from the past 10 years and prioritizing English-language publications. Papers describing CEAs were prioritized for inclusion. Congress presentations that included CEA were included based on expert knowledge and ability to retrieve poster and/or oral presentations.

**Results:**

31 CEAs comparing enhanced vaccines to standard-dose trivalent/quadrivalent vaccines (SD-TIV/SD-QIV) were analyzed, 15 comparing HD-TIV/HD-QIV with SD-TIV/SD-QIV and 17 comparing aTIV/aQIV with SD-TIV/SD-QIV. Enhanced vaccines were consistently cost-effective vs SD-TIV/SD-QIVs, despite diversity of model types, vaccine acquisition price, rVE estimate used, influenza-associated costs, and study perspective used. CEA results were inconsistent when the enhanced vaccines were compared with each other. The key driver of the latter was the different rVE approaches considered. While observational studies on vaccine effectiveness used in CEAs suggest effectiveness comparability between aTIV/aQIV and HD-TIV/HD-QIV, most studies show a lower acquisition price for aTIV/aQIV.

**Conclusion:**

Enhanced vaccines for older adults are more efficient compared to standard-dose vaccines. The adjuvanted influenza vaccine could reduce overall costs compared to high-dose vaccine if it has a lower acquisition price.

**Disclosures:**

**David Fisman**, CSL Seqirus: Advisor/Consultant **Norberto Giglio**, CSL Seqirus Inc.: Advisor/Consultant **Sergio Márquez-Peláez, MD**, CSL Seqirus Inc.: Advisor/Consultant **Van Nguyen, PhD**, CSL Seqirus Inc.: Advisor/Consultant|CSL Seqirus Inc.: Advisor/Consultant|CSL Seqirus Inc.: Honoraria **Maarten Postma, PhD**, CSL Seqirus Inc.: Advisor/Consultant|CSL Seqirus Inc.: Advisor/Consultant|CSL Seqirus Inc.: Honoraria **Andrea Pugliese, PhD**, CSL Seqirus Inc.: Advisor/Consultant **Jesús Ruiz-Aragón, MD**, CSL Seqirus Inc.: Advisor/Consultant **Analia urueña, n/a**, CSL Seqirus Inc.: Advisor/Consultant|CSL Seqirus Inc.: Advisor/Consultant **Joaquin F. Mould-Quevedo, PhD**, CSL Seqirus Inc.: Employee|CSL Seqirus Inc.: Employee|CSL Seqirus Inc.: Stocks/Bonds|CSL Seqirus Inc.: Stocks/Bonds

